# Immobilization of planktonic algal spores by inkjet printing

**DOI:** 10.1038/s41598-019-48776-z

**Published:** 2019-08-26

**Authors:** Hwa-Rim Lee, Sang Mok Jung, Sejeong Yoon, Woong Hee Yoon, Tae Hee Park, Seongju Kim, Hyun Woung Shin, Dong Soo Hwang, Sungjune Jung

**Affiliations:** 1 Department of Creative IT Engineering, Pohang University of Science and Technology (POSTECH), Pohang, 37673 Republic of Korea; 2 Department of Life Science and Biotechnology, Soonchunhyang University, Asan, 31538 Republic of Korea; 3 School of Interdisciplinary Bioscience and Bioengineering, Pohang University of Science and Technology (POSTECH), Pohang, 37673 Republic of Korea; 4 Division of Integrative Biosciences and Biotechnology, Pohang University of Science and Technology (POSTECH), Pohang, 37673 Republic of Korea; 5 Department of Mechanical Engineering, Pohang University of Science and Technology (POSTECH), Pohang, 37673 Republic of Korea

**Keywords:** Biotechnology, Ocean sciences

## Abstract

The algal cell immobilization is a commonly used technique for treatment of waste water, production of useful metabolites and management of stock culture. However, control over the size of immobilized droplets, the population of microbes, and production rate in current techniques need to be improved. Here, we use drop-on-demand inkjet printing to immobilize spores of the alga *Ecklonia cava* within alginate microparticles for the first time. Microparticles with immobilized spores were generated by printing alginate-spore suspensions into a calcium chloride solution. We demonstrate that the inkjet technique can control the number of spores in an ejected droplet in the range of 0.23 to 1.87 by varying spore densities in bioink. After the printing-based spore encapsulation, we observe initial sprouting and continuous growth of thallus until 45 days of culture. Our study suggest that inkjet printing has a great potential to immobilize algae, and that the ability to control the number of encapsulated spores and their microenvironments can facilitate research into microscopic interactions of encapsulated spores.

## Introduction

Immobilization of algal cells in polymeric hydrogels has a range of applications. Immobilized algal cells can be used for effluent treatment to remove nutrients, metals and industrial pollutants^1,2^. Entrapped algal cells can also be used to generate metabolite production^3,4^, measure toxicity^5,6^, preserve cells by freezing^7^, and to manage stock cultures^8^. The technique has also enabled improvement of metabolism, function, and growth of immobilized algal cells^9,10^. Methods to entrap microorganisms in hydrogel particles include conventional dripping of cell suspension into a receptacle that contains hardening solution^11^; extrusion dripping^12^; gravity-driven dripping^13^; and suspension spraying^14^. All of these methods are either slow or not allow sufficient control over the size of droplets, their content of microbes or production rate. A practical method would overcome these drawbacks.

*Ecklonia cava* is an edible, perennial marine brown alga (Laminariales, Phaeophyta) that grows in the ocean around South Korea and Japan^15^. *E. cava* is a major source of alginate, which is used for foods, pharmaceuticals, therapeutics and tissue engineering^16,17^. An extract of *E. cava*, phlorotannin, has been evaluated for ability to suppress tumor growth^18^ and as an antioxidation^19^. Therefore, *E. cava* has wide applications in science and industry. However, populations of *E. cava* are decreasing around east Asia^20^.

Drop-on-demand (DOD) inkjet printing is extensively used in various fields such as bioprinting^21,22^, printed electronics^23–25^ and 3D fabrication^26,27^. The DOD piezoelectric inkjet printing uses a piezoelectric actuator in the channel of the nozzle of piezoelectric inkjet printer. A voltage pulse reduces the volume of a chamber that contains ink, so some is ejected as a droplet^28^. Piezoelectric inkjet printing can generate droplets of size 1–100 pL at >10 kHz. The size of the ejected droplet can be controlled by adjusting the input voltage pulse or by selecting an appropriate nozzle and be smaller than the diffusion limits of nutrients and metabolites in hydrogels (100–200 µm)^12^. The small size of microparticles can minimize the inhibitory effect during the growth of the entrapped algal cells^29^. Owing to the ability to eject small volume of ink, inkjet printing has been used to encapsulate macromolecules^30^, drugs^17^ and mammalian cells^31,32^.

**Figure 1:**
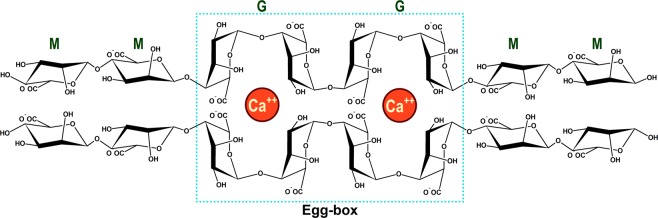
Schematic of “Egg-box” structure in alginate hydrogel crosslinked with calcium ions.

Alginate, one of the major polysaccharides in seaweeds and bacterial biofilms, is a copolymer of β-D- mannuronic acid (M block) and α-L-guluronic acid (G block). Alginate can form a hydrogel in the presence of calcium (Ca^2+^) or magnesium (Mg^2+^) ions. Especially G blocks in alginate contributes to form strong and reversible crosslinking networks so called “egg-box structure” (Fig. 1)^33,34^. Owing to the unique egg-box structure, alginate has been used for various coating applications in cosmetics, food, biomedical/pharmaceutical purposes, and Li-ion batteries^34–36^. Since alginates are mainly isolated from microalgae including *E. cava*, alginate was selected as a polymer for immobilizing *E. cava* spores with the inkjet printing technique.

The objective of this work is to prove the capability of the inkjet printing technique with alginate for immobilization of algal spores of *E. cava*. We measured the viscosities of alginate-spore suspensions and assessed the long-term stability of droplet ejection and jetting characteristics to evaluate inkjet-printability of the suspensions. Then, we monitored the numbers of spores in droplets to quantify the controllability of delivery. Finally, the growth of the immobilized spores in PESI/GeO_2_ medium was quantified to reveal a compatibility of the immobilization technique of algal spores.

## Materials and Methods

### Sodium alginate-spore suspension preparation

Sodium alginate was purchased from MP Biomedical LLC, USA. Reproductive thalli of *E. cava* were collected from the coast of Jeju Island, Korea, then shipped to the laboratory in an ice box. The thalli were washed with tap water to remove salt, sand and microorganisms. The middle part of thallus that includes sporangium was collected and the pieces were placed in sterilized seawater to liberate spores. The spore suspension was filtered using absorbent cotton. 2% (w/v) sodium alginate solution from brown algae (Sigma Aldrich, USA) was autoclaved for 20 min at 120 °C, cooled at room temperature and filtered using 0.45-µm syringe filter. The solution was mixed with the spore suspension to prepare a final 0.5% (w/v) sodium alginate solution with concentrations of 0.125 × 10^6^, 0.500 × 10^6^ or 2.00 × 10^6^ cells ml^−1^.

### Viscosity measurement

Shear viscosities of the suspensions were measured using a cone-plate rotational viscometer (DV2T, Brookfield AMETEK, US) at room temperature. The viscometer has a rotating cone with a radius of 2.4 cm and a stationary plate. Sodium alginate-spore suspension (0.5 ml) was placed between the plate and the cone. Shear rates between 50 s^−1^ and 1,500 s^−1^ were applied for 60 s; the viscosities were derived by averaging data of the final 10 s (*n* = 3).

### Inkjet printing of sodium alginate-spore suspensions

A DOD inkjet printing system (Jetlab II, MicroFab Technologies, Inc., USA) was used to print the sodium alginate-spore suspensions. A piezoelectric inkjet nozzle with a diameter of 80 µm was used and voltage pulse was applied at ±80 V in bipolar mode. The wave pulse had a rise time of 6.0 µs, dwell time of 13 µs, fall time of 9.0 µs, and echo time of 22 µs. Images of jet formation were acquired every 30 µs by a stroboscopic CCD camera equipped in the printing system, and a short-duration LED light.

Arrays of printed sodium alginate-spore suspensions were generated on glass slides. The drop spacing of the arrays was 400 µm and stage speed was 50 mm s^−1^. Printed arrays were observed under an optical microscope and the average number of spores per drop was calculated (*n* = 30).

### Fabrication and culture of spore-immobilized alginate microparticles

Sodium alginate-spore suspensions were printed using the inkjet printing system, the the printed drops were collected in a 35-mm Petri dish containing 1% (w/v) dissolved CaCl_2_ in seawater. The seawater had been autoclaved for 20 min at 120 °C, cooled at room temperature and passed through a 0.45-µm filter. Printing was conducted at an ejection frequency of 1,000 Hz for 30 min. The solution was strirred using a 1-cm long magnetic stirring bar to prevent aggregation of the hydrogel particles. After printing, 70 µl of generated microparticle suspension was mixed with 200 µl of PESI medium^37^ supplemented with GeO_2_ and delivered into each well of 96-well plates. The samples were incubated at 15 °C, light intensity of 30 μmol m^−2^ s^−1^, and diel cycle of 10 h light: 14 h dark. Thallus length of gametophytes of *E. cava* was measured under a microscope on days 3, 8, 14 and 21 of culture. The number of measured gametophytes of *E. cava* was different according to the used sodium alginate solutions with concentrations of 0.125 × 10^6^, 0.500 × 10^6^ or 2.00 × 10^6^ cells ml^−1^. (*n*_*0.125*_ = 7, *n*_*0.500*_ = 15, *n*_*2.00*_ = 20).

### Statistical analysis

All quantitative data are presented as the mean ± standard deviation. A one-way analysis of variance (ANOVA) with a Bonferroni post-hoc test was performed using Origin 2016 (Origin Lab, MA, US). *p*-values < 0.05 were considered to be statistically significant.

## Result and Discussion

### Strategy for fabrication of spore-immobilized alginate microparticles

**Figure 2:**
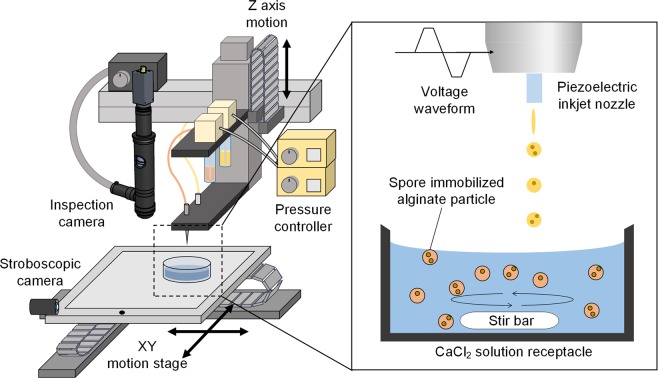
Schematic of system based on the piezoelectric inkjet printing technique to generate spore-immobilized microparticles.

Figure 2 illustrates the piezoelectric drop-on-demand inkjet printing system to generate alginate microparticles containing spores. When a voltage pulse is applied to a piezoelectric actuator of an inkjet nozzle, the nozzle ejects a series of picoliter droplets of sodium alginate-spore suspensions, which sink into a CaCl_2_ solution in a receptacle. As soon as a droplet impacts on the surface of the solution, Ca^2+^ enters guluronate blocks of alginate molecule, thus forming junctions with adjacent guluronate blocks^38^. As a result, alginate-based droplets are crosslinked and transformed into hydrogel microparticles that encapsulate spores. The solution in the receptacle was magnetically stirred to prevent aggregation of hydrogel particles (Supplementary Fig. 1).

### Viscosities and jet formations of sodium alginate-spore suspensions

**Figure 3:**
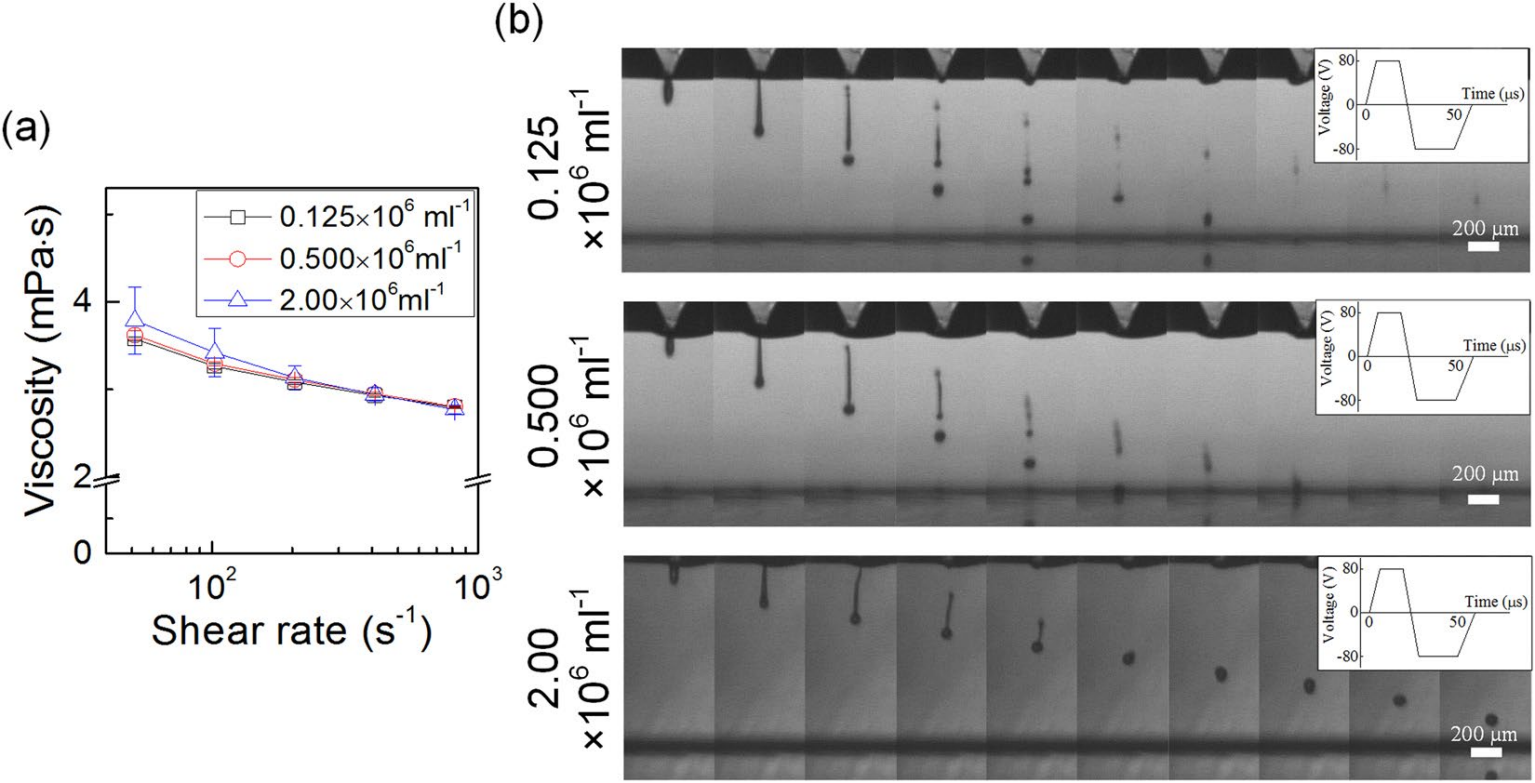
(**a**) Viscosities of sodium alginate-spore suspensions with different *E. cava* spore densities. (**b**) Jet formation images driven by the same input voltage (inset).

Viscosities of sodium alginate-spore suspensions was measured in the range of shear rate between 5 × 10^1^ and 10^3^ s^−1^ to examine their printability. All of the suspensions had viscosity <20 mPa∙s, which is suitably low for stable printing (Fig. 3a). The spore density did not significantly affect viscosities due to the very small size of spore (several microns) and the low range of spore densities (0.125 × 10^6^ ml^−1^ to 2.00 × 10^6^ ml^−1^). Then, we observed their jetting behaviors for 2 hours. No clogging of the nozzle was observed during the experiments. Their representative high-speed images of jet formation are shown in Fig. 3b. With the same driving waveform and the negligible difference in shear viscosity, drop velocity was observed to decrease as spore density increased. We speculate that the naturally secreted component by *E. cava* spores inside the suspensions might slightly increase the elasticity of bioink.

### Average number of spores per drop

**Figure 4:**
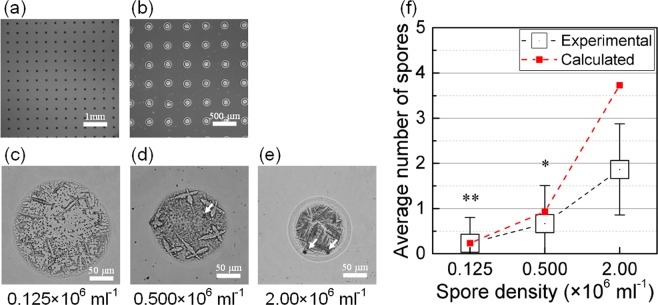
Printing of dot array pattern to count the average spore number per droplet. Microscope images of the dot array with 20x (**a**) and 40x (**b**) magnifications. Representative images of dot of sodium alginate-spore suspensions with 0.125 × 10^6^ cells ml^−1^ (**c**), 0.500 × 10^6^ cells ml^−1^ (**d**) and 2.00 × 10^6^ cells ml^−1^ (**e**). The size of ejected drops on glass substrate are around 200 μm (**c**), 150 μm (**d**) and 130 μm (**e**). White arrows indicate spores. (**f**) Experimental and calculated average number of spores per droplet with respect to spore densities (*p < 10^−4^, **p < 10^−5^, compared to the value obtained at 2.00 × 10^6^ cells ml^−1^).

In order to quantify the controllability of delivery of spores, we investigated the number of spores in a droplet by printing dot arrays of sodium alginate-spore suspensions onto the glass substrate without crosslinking. Then, the number of spores in a drop was counted using microscope (Fig. 4a,b). Examination of the dot array showed increase in the average number of spores per drop as spore densities increased: 0.23 at 0.125 × 10^6^ ml^−1^, 0.67 at 0.500 × 10^6^ ml^−1^, and 1.87 at 2.00 × 10^6^ ml^−1^ (Fig. 4f), and decrease in the size of dots as spore density increased (Fig. 4c–e). However, the average number of spores per drop did not increase as much as the spore density of the suspension did. The spore population may have increased the viscosity and elasticity of the suspension and reduced ejection volume of ink from the nozzle, as was also observed in a previous study^39^. This ability to control the number of encapsulated spores and their microenvironments demonstrates that the piezoelectric inkjet printing technique has possible uses as a method to study microscopic interactions of encapsulated spores.

### Generation and culture of *E. cava* spores immobilized in alginate microparticles

**Figure 5:**
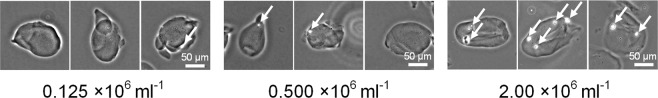
Microscopic images of spore-immobilized microparticles with different spore densities.

Microparticles were generated using inkjet printing system with a receptacle containing CaCl_2_ crosslinking solution as shown in Fig. 2. In order to monitor the morphology of spore-laden microparticles, they were observed using a microscope. The spores of *E. cava* were immobilized inside alginate microparticles (Fig. 5, white arrows). The crosslinking of alginate with calcium ion was proceeded quickly from the boundary of the printed droplet, the loss of spores is not considered during this process and the expected number of encapsulated spores are identical to that in Fig. 4. The number of spores immobilized within the microparticles increased with the spore density of the suspensions. The shape of the formed microparticles were elliptical because of vigorous agitation by magnetic stirrer.

**Figure 6:**
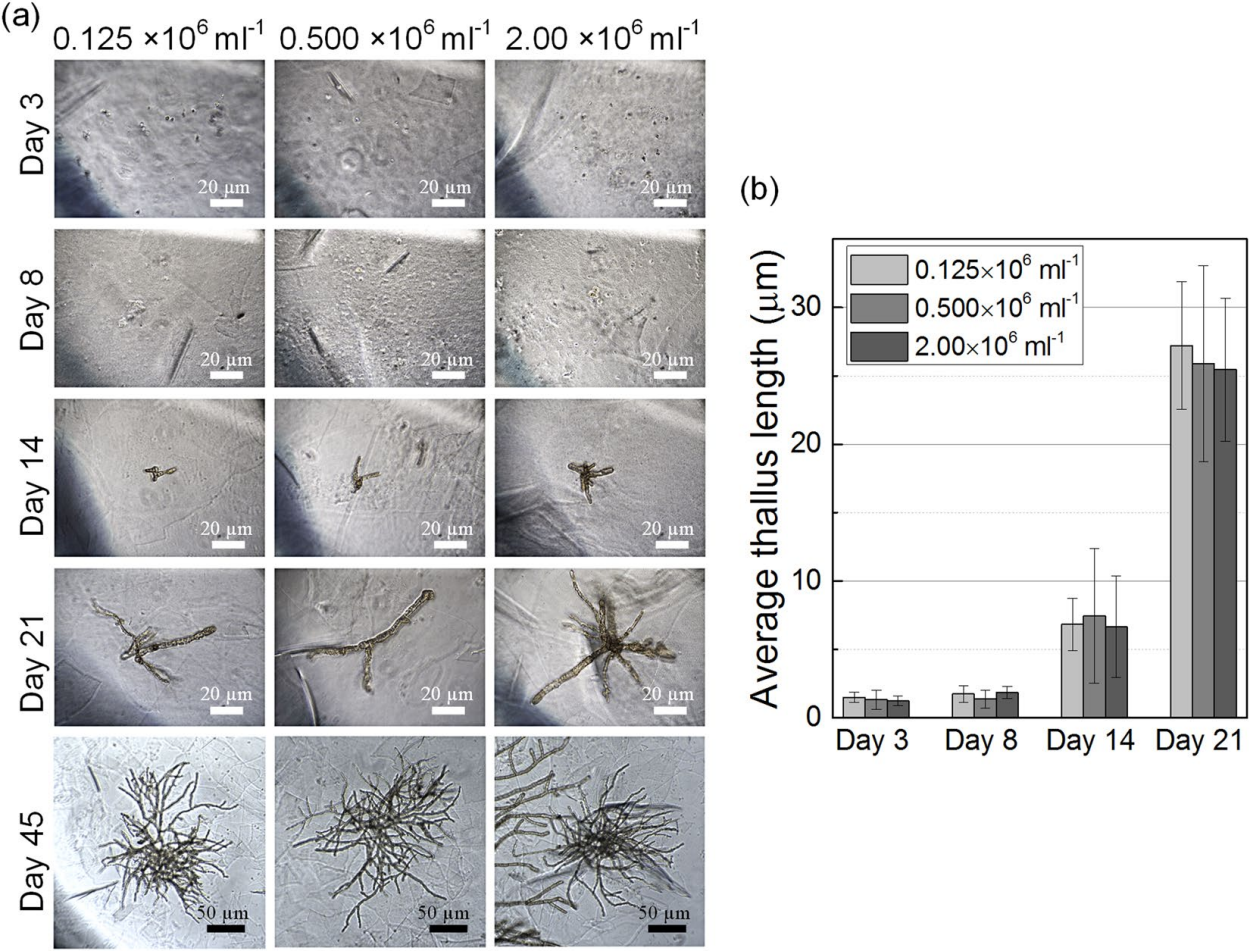
(**a**) Representative images of *E. cava* gametophytes immobilized in alginate microparticles with different spore densities. (**b**) Thallus length of *E. cava* gametophytes immobilized within alginate microparticles.

Sprouting and growth of the entrapped spores were monitored for 45 d to prove a compatibility of the immobilization technique of algal spores. Spores immobilized in microparticles remained dormant until day 8 (Fig. 6), then a thallus began to grow. Average thallus length reached ~10 µm on day 14, and ~30 µm on day 21. After 45 days of culture, *E. cava* showed a bush-like morphology. The growing *E. cava* showed the gametophyte phenotype because the spores had been collected from sporophytes^10^. The results show that inkjet printing has applications for immobilization of algal cells. Furthermore, the growth trend was not significantly affected by spore density. This result indicates that *E. cava* spores at low to moderate density of within hydrogel microparticles could not significantly influence each other’s thallus morphology. The range of the spore density at which such influence occurs should be determined in future studies.

## Conclusion

In this study, piezoelectric inkjet printing technology was used to immobilize spores of *E. cava* in sodium alginate suspensions. Viscosities and drop-formation behaviors demonstrated that they were suitable inks for the inkjet printing. The spatial density of spores may affect the viscosity and the elasticity of sodium alginate-spore suspension, and thereby affect jetting formation and lower-than-expected average number of spores per droplet. The printing technique allowed fabrication of spore-immobilized alginate microparticles. Culture of the microparticles yielded healthy gametophytes of *E. cava* without significant dependence on the spore density. These results show that inkjet printing has applications for immobilization of algal cells and single cell encapsulations.

## Supplementary information

Supplementary Figure 1
