# Supplementary material for: Immobilization of planktonic algal spores by inkjet printing
Source: Sci Rep. 2019 Aug 26;9:12357. doi: 10.1038/s41598-019-48776-z (PMC6710280; doi:10.1038/s41598-019-48776-z)
Supplement: Supplementary file 1 — Supplementary Figure 1 [file 41598_2019_48776_MOESM1_ESM.docx]

***Supplementary information for***

**Immobilization of planktonic algal spores by inkjet printing**

Hwa-Rim Lee^a,1^, Sang Mok Jung^b,1^, Sejeong Yoon^c^, Woong Hee Yoon^d^, Tae Hee Park^b^, Seongju Kim^e^, Hyun Woung Shin^b, *^, Dong Soo Hwang^d,*^, Sungjune Jung^a, c, d, e *^

^a^ Department of Creative IT Engineering, Pohang University of Science and Technology (POSTECH), Pohang, 37673, Republic of Korea

^b^ Department of Life Science and Biotechnology, Soonchunhyang University, Asan, 31538, Republic of Korea

^c^ School of Interdisciplinary bioscience and bioengineering, Pohang University of Science and Technology (POSTECH), Pohang, 37673, Republic of Korea

^d^ Division of Integrative Biosciences and Biotechnology, Pohang University of Science and Technology (POSTECH), Pohang, 37673, Republic of Korea

^e^ Department of Mechanical Engineering, Pohang University of Science and Technology (POSTECH), Pohang, 37673, Republic of Korea

^1^ Equally contributed

*Email address: [hwshin@sch.ac.kr](mailto:hwshin@sch.ac.kr), [dshwang@postech.ac.kr](mailto:dshwang@postech.ac.kr), [sjjung@postech.ac.kr](mailto:sjjung@postech.ac.kr)


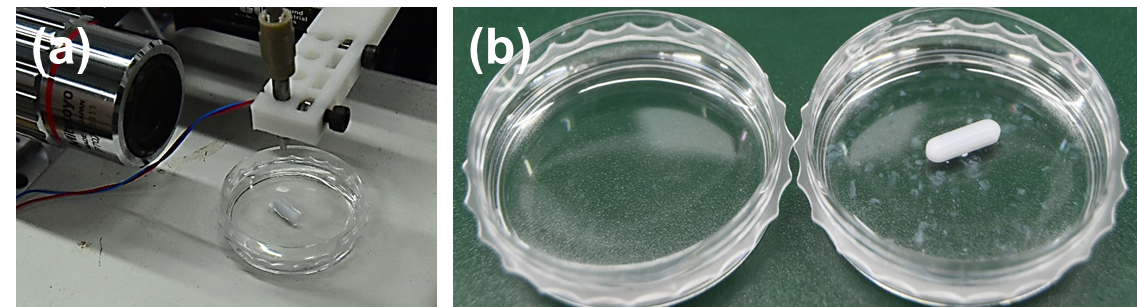


**Fig. S1.** The real image of inkjet printing-based spore immobilization. (a) The image of an ongoing spore printing process. (b) Photo images of 0.5% alginate bioink before and after crosslinking.
